# Haematological values in a healthy adult population in Yaoundé, Cameroon

**DOI:** 10.4102/ajlm.v8i1.852

**Published:** 2019-10-31

**Authors:** Martine E. Oloume, Abas Mouliom, Bernard F. Melingui, Suzanne Belinga, Julie S. Nana, Mathurin Tejiokem, Francoise N. Sack, Jeanne Manga, Annie R. Epote

**Affiliations:** 1 Haematology Laboratory, Centre Pasteur, Yaoundé, Cameroon; 2 Medical Analysis Department, Centre Pasteur, Yaoundé, Cameroon; 3 Haematology and Immunology Department, Catholic University of Central Africa, Yaoundé, Cameroon; 4 Epidemiology Department, Centre Pasteur, Yaoundé, Cameroon; 5 Haematology Department and Blood Bank, Central Hospital, Yaoundé, Cameroon; 6 Quality Management, Centre Pasteur, Yaoundé, Cameroon

**Keywords:** complete blood cell count, reticulocyte count, locally derived ranges

## Abstract

**Background:**

Haematological values derived from local populations are useful in laboratories to improve diagnoses for local patients. In Cameroon, these data are not yet available. Moreover, there is great variation in baseline parameters pertaining to full blood cell count among medical laboratories.

**Objectives:**

This study aimed to determine values for the complete blood cell count of a healthy adult Cameroonian population for use in locally derived ranges in our medical laboratories.

**Methods:**

A cross-sectional study was conducted among blood donors attending three blood banks in Yaoundé from November 2015 to September 2016. We expected to obtain at least 120 venous blood samples from both men and women. Tests were performed for (1) HIV, (2) complete blood cell count, (3) hepatitis B virus, (4) malaria, (5) syphilis, (6) C-reactive protein and (7) hepatitis C virus.

**Results:**

We enrolled 294 healthy participants (161 men, 133 women) aged 18 to 55 years. The median haemoglobin concentration was 135 g/L in men and 114 g/L in women (*p* < 0.001). The median reticulocyte count was 60 × 10^9^/L in men and 40 × 10^9^/L in women (*p* < 0.001). Significant variation by sex was observed for the platelet count. The median white blood cell count was 4.1 × 10^9^/L in men and 4.6 × 10^9^/L in women (*p* = 0.008).

**Conclusion:**

This study provides locally derived ranges for complete blood cell and reticulocyte counts for a healthy adult population in Yaoundé, Cameroon. These results can be used pending larger studies.

## Introduction

A complete blood cell count is the most current and simplest method for assessing medullary function and haematopoiesis. It is a routine analysis that is very useful in medical practice; this test can reveal very diverse pathologies. A proper interpretation is therefore essential to guide the diagnosis and request an additional medical analysis. Until now, haematological reference values have not been established for the Cameroonian population. Normal values used in our laboratories are provided from the literature or instrument manuals. These values usually come from industrialised countries and are not always suitable for our context. Cameroon has a heterogeneous population consisting of 250 ethnic groups that can be classified into five main groups: (1) southern tropical forest peoples, (2) western highlanders, (3) coastal tropical forest peoples, and the (4) Kirdi and (5) Fulas ethnic groups. Yaoundé is a multicultural city in which these communities can be found. Factors such as age, sex and race in addition to environment can significantly influence blood count parameters.^1,2,3^ The Clinical and Laboratory Standards Institute recommends that laboratories develop their own local values.^4^ Furthermore, there is a great variability in reference values between medical analysis laboratories in Cameroon. This study was intended to determine the values of a complete blood cell count in a healthy adult population for better quality diagnosis, which could in some cases prevent unnecessary testing.

## Methods

### Ethical considerations

Ethical approval was obtained from the Cameroon National Ethical Committee of Research for Human Health (permit number: 2016/01/701/CE/CNERSH/SP). Every participant gave written informed consent before the sample collection.

### Study population and sampling strategy

This was a cross-sectional study conducted from November 2015 to September 2016. The target population constituted blood donors, because they were submitted to a rigorous medical screening that took into account several criteria for detecting pathology, thus ensuring a healthy population for the study. The recruitment sites were the blood banks of the most frequented hospitals, including the University Teaching Hospital of Yaoundé, the Yaoundé Central Hospital, and the Hospital Centre of Essos. A stratified and clustered sampling strategy enabled us to divide the population into two groups (men and women) and collect around 50 samples per site and per sex. According to the Clinical and Laboratory Standards Institute,^4^ a study requires a minimum of 120 samples in each group in order to obtain valid reference values. A survey was administered before the blood donation that enabled the selection of participants and the collection of socio-demographic data. All of the eligible blood donors who gave their informed consent were included in the investigation. People who met one or more of the following criteria were excluded: (1) currently sick or being followed up for a known pathology, (2) pregnant women, (3) undergoing treatment for any disease, (4) history of blood donation or transfusion within the immediate past 4 months, (5) regular smokers and (6) hospitalisation within the immediate past month. In addition, we excluded people with a positive serology result for any one of the serological tests carried out at the blood bank, including HIV, syphilis, hepatitis B virus, hepatitis C virus, positivity to blood parasites through a thick or thin blood film, platelet aggregates, C-reactive protein > 6 mg/L, or inflammatory syndrome, as well as people with hyperthermia (˃ 37.8 °C) or hypothermia (< 35 °C) as determined by thermometer as a result of temperature control-related conditions.

### Laboratory analysis

For each participant, blood was drawn from a superficial vein in the upper limb using venepuncture sampling. This allowed the collection of 5 mL of blood in an ethylene diamine tetraacetic acid tube, which was used for full blood count, reticulocyte count and blood parasite analysis by a thick and thin blood film, and 4 mL of blood in a tube without anticoagulant for C-reactive protein analysis. Those tests were performed at the laboratory of Centre Pasteur Cameroon. Samples were stored at room temperature in an air-conditioned room (20 °C – 23 °C). Samples were transported in a cooler containing a tube rack to avoid thermal or mechanical shocks that could damage cells and cause haemolysis. Analyses were done within 6 hours of blood collection. A peripheral blood smear and a thick film were prepared for every sample.

Serological tests for HIV, syphilis, hepatitis B virus, and hepatitis C virus were carried out by every blood bank. Human immunodeficiency virus (HIV) antibodies were detected by two rapid tests that detect both HIV-1 and HIV-2 infections: (1) the ImmunoComb HIV 1&2 BiSpot (Orgenics, Courbevoie, France) and (2) the Determine HIV-1/2 (Abbott Laboratories, Chicago, Illinois, United States), an immunochromatographic assay. Samples reactive to only one of the two rapid testing procedures were considered indeterminate and further tested using an ELISA method. Hepatitis B virus was detected using a one-step, immunoassay-based DIASpot HBsAg test kit (DIASpot Diagnostics, Jawa Barat, Indonesia) for qualitative detection of serum hepatitis B surface antigen. IgG antibodies to hepatitis C virus were detected using DIASpot HCV-Ab test strips (DIASpot Diagnostics, Jawa Barat, Indonesia), an immunochromatographic assay. Syphilis was diagnosed using the venereal disease research laboratory test and the *Treponema pallidum* hemagglutination assay test (Omega Diagnostic, Alva, Scotland, United Kingdom).

The full blood and reticulocyte counts were analysed using Pentra DX Nexus (Horiba-ABX, Montpellier, France). This analyser allows for the differentiation and quantification of 28 parameters of a haematopoietic population with five reference methods of measurement: (1) flow cytometry, (2) cytochemistry, (3) impedance, (4) absorbance and (5) optical cytometry. The reticulocyte count was performed using fluoro-flow cytometry with orange thiazol.

### Quality control

Levels of internal quality controls (Horiba-ABX difftrol, ABX minotrol reti) were analysed daily on three samples: (1) low, (2) normal and (3) high. The performance of the laboratory was checked based on the external proficiency testing program with Biology Prospective (Villers-lès-Nancy, France). The laboratory has been audited as per the World Health Organization Regional Office for Africa’s Laboratory Improvement Process Towards Accreditation (SLIPTA) Checklist, and has achieved a four-star ranking on the SLIPTA Tier of Recognition of Laboratory Quality Management (valid: September 2017 to September 2019).

### Statistical analysis

Data were registered in the Excel 2013 software (Microsoft Corp., Redmond, Washington, United States) and analysed with Epi Info 7 (Centers for Disease Control and Prevention, Atlanta, Georgia, United States) and SPSS 19 software (IBM Corp., Chicago, Illinois, United States). The data sets were not tested for a Gaussian distribution, which was taken into account in the data analysis. For each parameter, the standard deviation and the median with the 2.5th percentile and 97.5th percentile were calculated. The 2.5th percentile was used to define the lower reference limit, while the 97.5th percentile defined the upper one. A Kruskal–Wallis test for the two groups was used to correlate parameters according to sex. The threshold for significance was set at *p* < 0.05 for all statistical analyses.

## Results

### Demographic characteristics

**FIGURE 1:**
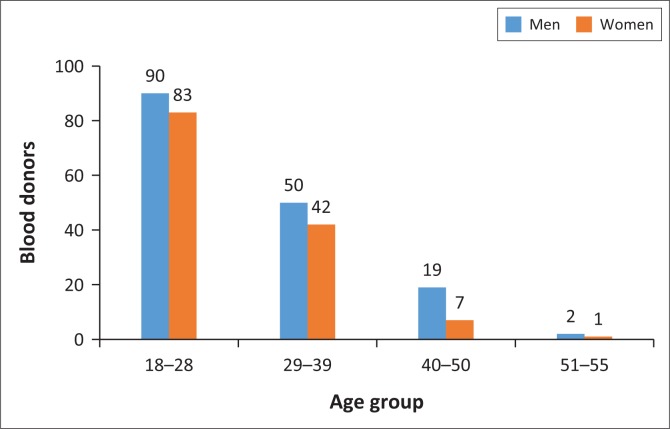
Distribution of study participants by age group, Yaoundé, Cameroon, November 2015 – September 2016.

The total number of people approached and asked to participate in the study was 375. After applying the exclusion criteria, 294 participants (161 men and 133 women) were maintained in order to determine normal values for the local population. A positive serology result for any one of the serological tests carried out at the blood bank, and positivity to blood parasites were major reasons participants were excluded (Table 1). The sex ratio was 1:12. Their ages ranged from 18 to 55 years for men and 18 to 53 years for women (Figure 1). Most participants identified as southern forest peoples (Table 2). The majority of the population (59%) was between age 18 and 28 years and 31% was between age 29 and 39 years. In terms of blood donation, 61% of the population had never donated blood before. The distribution was approximately equal between the three sites. Once the exclusions had been applied, around 45 donors of each sex were included per site.

**TABLE 1:** Exclusion criteria applied to the study population in Yaoundé, Cameroon, November 2015 – September 2016.

Exclusion criteria†	Men (*N* = 222)	Women (*N* = 153)	Total (*N* = 375)
HIV	4	1	5
Hepatitis B	17	3	20
Hepatitis C	10	1	11
Syphilis	8	2	10
Blood parasites (microfilariae, malaria)	9	6	15
C-reactive protein ˃ 6 mg/L	33	11	44
Platelet aggregates	1	3	4
Smokers ˃ 5 cigarettes/day	2	0	2
Population	222	153	375
Population after exclusion criteria were applied	161	133	294

† , Many participants met several exclusion criteria at the same time. For example, some donors presented an increased C-reactive protein and a positive serology test or a positive blood parasite test and therefore appear more than once as exclusions.

**TABLE 2:** Study population by main ethnic groups, Yaoundé, Cameroon, November 2015 – September 2016.

Ethnic groups	Frequency	Percentage
Southern forest peoples	135	45.9
Kirdi	3	1.0
Coastal forest peoples	39	13.3
Western highlanders	108	36.7
Fulas	9	3.1
**Total**	**294**	**100.0**

### Red blood cell parameters

The median haemoglobin concentration was 135 g/L in men and 114 g/L in women and the difference was statistically significant (*p* < 0.001) (Table 3). A statistically significant difference between men and women was also noted for red blood cell and haematocrit values, with men having higher values than women. Normal local values were found for erythrocyte parameters; no differences between men and women were observed for mean corpuscular volume, the mean corpuscular haemoglobin concentration, the mean haemoglobin concentration, and the red blood cell distribution index. The reticulocyte count was higher in men compared with women and the difference was statistically significant.

**TABLE 3:** Erythrocyte parameters for men and women, Yaoundé, Cameroon, November 2015 – September 2016.

Parameter	Men (*N* = 161)		Women (*N* = 133)		*P*
	Median	2.5% – 97.5%	Median	2.5% – 97.5%	
Red blood cells (× 10^12^/L)	4.87	4.00–5.90	4.11	3.40–5.30	< 0.001
Haemoglobin (g/L)	135.00	110.00–160.00	114.00	98.90–137.00	< 0.001
Haematocrit (%)	41.80	34.60–47.61	35.61	29.70–42.00	< 0.001
MCH (pg)	28.20	22.80–33.40	28.10	23.22–31.20	0.890
MCHC (g/L)	323.00	299.00–353.00	321.00	300.00–342.00	0.054
MCV (fL)	86.00	70.00–97.00	87.00	72.00–96.00	0.470
RDI (%)	17.00	13.40–21.00	16.70	13.70–20.00	0.900
Reticulocytes (× 10^9^/L)	60.00	30.00–250.00	40.00	20.00–177.00	< 0.001

MCHC, mean corpuscular haemoglobin concentration; MCH, mean haemoglobin concentration; MCV, mean corpuscular volume; RDI, red blood cell distribution index; *N*, number.

### Platelet parameters

The number of platelets was higher in women (median: 243 × 10^9^/L) than in men (median: 211 × 10^9^/L) (Table 4). Neither the mean platelet volume nor the platelet distribution index differed by sex.

**TABLE 4:** Platelet parameters for men and women, Yaoundé, Cameroon, November 2015 – September 2016.

Parameter	Men (*N* = 161)		Women (*N* = 133)		*P*
	Median	2.5% – 97.5%	Median	2.5% – 97.5%	
Platelets (× 10^9^/L)	211.00	133.00–339.00	243.00	143.00–369.00	< 0.001
MPV (fL)	9.20	7.60–11.00	9.30	7.40–11.30	0.13
THT (%)	0.19	0.13–0.32	0.23	0.12–0.34	< 0.001
PDI (%)	16.10	11.90–21.30	16.30	11.50–21.80	0.37

MPV, mean platelet volume; THT, thrombocrit; PDI, platelet distribution index; *N*, number.

### White blood cell parameters

The median white blood cell count was 4.1 × 10^9^/L in men and 4.6 × 10^9^/L in women (Table 5). Statistically significant differences between men and women were found in the levels of neutrophils, eosinophils and monocytes. No differences between men and women were observed for lymphocytes or basophils.

**TABLE 5:** White blood cell parameters for men and women, Yaoundé, Cameroon, November 2015 – September 2016.

Parameter	Men (*N* = 161)		Women (*N* = 133)		*P*
	Median	2.5% – 97.5%	Median	2.5% – 97.5%	
White blood cell (× 10^9^/L)	4.10	2.60–6.81	4.60	2.80–6.70	0.008
Neutrophils (× 10^9^/L)	1.60	0.92–3.00	1.98	1.01–3.53	0.002
Eosinophils (× 10^9^/L)	0.20	0.05–0.98	0.15	0.12–0.42	< 0.001
Basophils (× 10^9^/L)	0.08	0.03–0.22	0.08	0.03–0.29	0.48
Lymphocytes (× 10^9^/L)	1.71	1.03–3.13	1.89	1.06–2.90	0.11
Monocytes (× 10^9^/L)	0.26	0.11–0.54	0.32	0.14–0.65	0.003

## Discussion

This study took place in Yaoundé, a cosmopolitan town that is located in the central region of the country. It consists of a number of hills that have an altitude of 600 m to 700 m above sea level, and a plateau. The highest mountain is 1221 m in altitude, where few people live. At an altitude above 2000 m, the effect that this has on haematological values is mainly an increase in the haemoglobin level.^2^

Medical laboratories more often than not use data provided by an analysis or selected from literature data according to whether they consider these sources to be a relevant reference. Several reviews have confirmed that reference values obtained in Western countries differ significantly from African populations.^2,3^ During studies to obtain reference values, ensuring a healthy study population is a painstaking and expensive exercise because medical tests must be carried out to confirm this. Blood donors could be considered an accessible population that was submitted to a rigorous medical selection. In addition to the blood donation serology, we carried out blood parasite analysis and eliminated all subjects presenting an inflammatory syndrome through C-reactive protein analysis.

In most cases, the results correlate with studies carried out in other African countries.^7,8,9,10^ Table 6 provides haematological locally derived ranges from our study compared with sources from other studies. The difference obtained between men and women during the red blood cell count and haemoglobin concentration determination is a confirmed fact. Men have higher values than women. In our study, the haemoglobin concentration ranges from 110 g/L to 160 g/L in men and 98.9 g/L to 137 g/L in women. In a study carried out by Dossoh et al. in Ghana, the same results were observed in men with values ranging from 80 g/L to 140 g/L in women.^11^ These variations have been attributed to factors such as the effect of the androgen hormone on erythropoiesis and menstrual blood loss in women.^2^ In similar African studies,^7,9^ the haemoglobin concentration values were lower compared to data from a population in France.^12^ The prevalence of sickle cell disease in Cameroon is around 2%.^13^ One of the limitations of the study is that we did not check for haemoglobin abnormalities among participants.

**TABLE 6:** Haematological locally derived ranges from our study compared with sources from other studies.

Parameters	Our study	Ghana^11^	Togo^7^	Kenya^15^	France^12^
**Haemoglobin (g/L)**					
Male	110.00–160.00 (135.00)	113.00–164.00 (139.00)	100.00–184.00 (151)	114.00–169.00 (14.2)	134.00–167.00
Female	98.90–137.00 (114.00)	88.00–144.00 (123.00)	103.00–171.00 (130)	80.00–142.00 (121.0)	115.00–149.00
**Red blood cells (×10^12^/L)**					
Male	4.00–5.90 (4.87)	3.79–5.96 (4.84)	3.30–6.40 (5.0)	4.30–6.50 (5.3)	4.53–5.79
Female	3.40–5.30 (4.11)	3.09.00–5.30 (4.32)	3.10–6.00 (4.5)	3.40–5.70 (4.5)	4.01–5.19
**Haematocrit (%)**					
Male	34.60–47.61 (41.80)	33.2–50.5 (42.20)	28.00–54.00 (42.8)	32.60–51.50 (41.7)	39.20–48.60
Female	29.70–42.00 (35.61)	26.4–45.0 (36.90)	28.00–47.00 (38.1)	23.20–44.30 (35.8)	34.40–43.90
**MCH (pg)**					
Male	22.80–33.40 (28.20)	22.7–33.5 (29.10)	26.00–36.00 (29.7)	NA	27.30–32.80
Female	23.22–31.20 (28.10)	22.3–33.6 (28.40)	25.00–37.00 (29.3)	NA	24.40–32.10
**MCHC (g/L)**					
Male	299.00–353.00 (323.00)	306.00–360.00 (331.00)	290.00–390.00 (351.0)	NA	324.00–363.00
Female	300.00–342.00 (321.00)	304.00–365.00 (331.00)	300.00–410.00 (351.0)	NA	319.00–358.00
**MCV (fL)**					
Male	70.00–97.00 (86.00)	70.00–98.00 (88.00)	80.00–99.00 (85.0)	55.00–98.00 (80.0)	79.60–94.00
Female	72.00–96.00 (87.00)	73.00–96.00 (86.00)	80.00–95.00 (84.0)	60.00–94.00 (79.0)	74.70–94.20
**Reticulocytes (×10^9^/L)**					
Male	30.00–250.00 (60.00)	NA	NA	NA	NA
Female	20.00–177.00 (40.00)	NA	NA	NA	NA
**Platelets (×10^9^/L)**					
Male	133.00–339.00 (211.00)	88.00–352.00 (208.00)	120.00–443.00 (239.0)	102.00–307.00 (201.0)	172.00–398.00
Female	143.00–369.00 (243.00)	89.00–408.00 (224.00)	150.00–436.00 (247.0)	88.00–439.00 (220.0)	185.00–445.00
**White blood cell (×10^9^/L)**					
Male	2.60–6.81 (4.10)	3.50–9.20 (5.50)	1.90–10.10 (4.1)	2.50–7.40 (5.3)	4.09–11.00
Female	2.80–6.70 (4.60)	3.40–9.30 (5.30)	2.20–7.80 (4.2)	3.30–9.70 (5.6)	4.02–11.40
**Neutrophils (×10^9^/L)**					
Male	0.92–3.00 (1.60)	1.50–5.90 (2.70)	0.50–5.40 (1.6)	0.80–3.90 (2.0)	1.78–6.90
Female	1.01–3.53 (1.98)	1.40–5.50 (2.70)	0.50–4.40 (1.6)	1.30–3.80 (2.3)	1.75–7.50
**Eosinophils (×10^9^/L)**					
Male	0.05–0.98 (0.20)	NA	0.00–0.05 (0.2)	0.10–1.70 (0.5)	0.048–0.59
Female	0.12–0.42 (0.15)	NA	0.00–0.05 (0.2)	0.10–1.30 (0.4)	0.041–0.55
**Lymphocytes (×10^9^/L)**					
Male	1.03–3.13 (1.71)	1.20–5.20 (2.20)	1.10–4.03 (2.1)	1.00–3.50 (2.2)	1.34–3.91
Female	1.06–2.90 (1.89)	1.20–4.40 (2.10)	1.20–4.30 (2.2)	1.30–3.80 (2.2)	1.24–3.56
**Monocytes (×10^9^/L)**					
Male	0.11–0.54 (0.26)	0.20–1.40 (0.50)	0.05–0.80 (0.2)	0.20–0.90 (0.5)	0.22–0.77
Female	0.14–0.65 (0.32)	0.20–0.90 (0.40)	0.05–0.80 (0.2)	0.30–0.80 (0.5)	0.20–0.71
**Basophils (×10^9^/L)**					
Male	0.03–0.22 (0.08)	NA	NA	0.01–0.19 (0.04)	0.00–0.097
Female	0.03–0.29 (0.08)	NA	NA	0.00–0.20 (0.04)	0.00–0.085
**Population**					
Blood Donors					
Male (*n*)	161	-	1047	-	-
Female (*n*)	133	-	302	-	-
Urban					
Male (*n*)	-	316	-	-	-
Female (*n*)	-	308	-	-	-
Rural					
Male (*n*)	-	-	-	77	-
Female (*n*)	-	-	-	83	-
National					
Male (*n*)	-	-	-	-	19 393
Female (*n*)	-	-	-	-	13 526

Note: Reference ranges, 2.5% – 97.5%.

MCHC, corpuscular haemoglobin concentration; MCH, haemoglobin concentration; MCV, corpuscular volume; NA, not available (median values).

In an American study^14^ consisting of 166 black and 296 white patients, the average haemoglobin concentration was lower among Black patients. As stated elsewhere, our neutrophil results progress in the same direction, although the values are more elevated in the populations of Western countries^12^ compared to the African population.^7,15^ The reason that populations in Western countries have higher values of neutrophils has not been well elucidated. The theory of an excess of marginated neutrophil pools, that these blood cells would be found more in myeloid organs and less in peripheral blood, is often suggested. However, a previous study seemed to disprove this hypothesis.^16^

Our study demonstrates higher eosinophil values in men compared to women. The same result was observed in a study done in France.^17^ Many reviews define eosinophilia as exceeding an upper limit of about 0.6 × 10^9^/L for men and women.^18^ There is not supposed to be any ethnic variation in the normal eosinophil count and no physiological cause of an increased count has been offered. The most common causes of eosinophilia depend on socioeconomic and geographical factors. In the developing world, the most common cause is parasitic infection, whereas in the developed world, it is allergy.^18^ The overall prevalence of soil-transmitted helminthiasis was 25.12% and 10.71% for schistosomiasis in the central region of Cameroon in 2012.^19^ Those results concerned school-aged children, and the prevalence of these infections is probably lower in adults and in urban areas.^20^ The National Programme for the Control of Schistosomiasis and Soil-Transmitted Helminthiasis has made great strides in reducing the burden caused by these diseases on the Cameroonian people.

As far as platelets are concerned, we obtained higher values in women compared to men (*p* < 0.001). The same results were obtained in Togo and Uganda and in a United Kingdom study^7,9,21^ and in France.^12^ However, our values were lower compared to the United Kingdom and French studies. The platelet count seems to be lower among black people than in white people. The reticulocyte count is used to determine if the bone marrow is responding adequately to the body’s need for red blood cells. It helps to find the cause of anaemia and to classify hypo and hyper proliferative anaemias.^22,23^ A few studies in Africa have determined the normal ranges of reticulocytes. In our study, the reticulocyte count was significantly higher in men than in women. In a study done by Tarallo et al.,^24^ the observed ranges were also found to be different between men and women. Nevertheless, our limit values were higher than those found in a population in France.^24^ This finding is interesting, because it could mean that the interpretation of reticulocyte counts should be based on sex, similar to those for haemoglobin parameters.

### Conclusion

Haematological values of the local population are necessary in order to improve daily diagnosis. We conducted a small-scale pilot study due to limited financial support before launching a project. Although the size of our sample was smaller than in other studies, the median values of many parameters, especially haemoglobin and platelet counts, were comparable to the ones found in other studies conducted in Africa but different to those in the northern hemisphere countries. This study took place in the capital city, which is a cosmopolitan town. It would be more appropriate to use these results rather than the standards from foreign countries and to eliminate the variability in reference ranges between local laboratories.
